# Non-proteolytic ubiquitylation in cellular signaling and human disease

**DOI:** 10.1038/s42003-022-03060-1

**Published:** 2022-02-08

**Authors:** Yongrong Liao, Izabela Sumara, Evanthia Pangou

**Affiliations:** 1 Institut de Génétique et de Biologie Moléculaire et Cellulaire (IGBMC), Illkirch, France; 2 Centre National de la Recherche Scientifique (CNRS), UMR 7104 Strasbourg, France; 3 Institut National de la Santé et de la Recherche Médicale (INSERM), U964 Strasbourg, France; 4 Université de Strasbourg, Strasbourg, France

**Keywords:** Ubiquitylation, Mechanisms of disease

## Abstract

Ubiquitylation is one of the most common post-translational modifications (PTMs) of proteins that frequently targets substrates for proteasomal degradation. However it can also result in non-proteolytic events which play important functions in cellular processes such as intracellular signaling, membrane trafficking, DNA repair and cell cycle. Emerging evidence demonstrates that dysfunction of non-proteolytic ubiquitylation is associated with the development of multiple human diseases. In this review, we summarize the current knowledge and the latest concepts on how non-proteolytic ubiquitylation pathways are involved in cellular signaling and in disease-mediating processes. Our review, may advance our understanding of the non-degradative ubiquitylation process.

## Abstract (web summary)

Evanthia Pangou and co-authors review recent insights into the important roles of non-proteolytic ubiquitylation in cellular signaling as well as in physiology and disease.

## Introduction

Ubiquitin (Ub) was first discovered in 1975 by Gideon Goldstein^1^. It is a small and highly conserved protein that is expressed in all eukaryotic cells^2,3^. There are four human genes encoding Ub precursors: RPS27A, UBA52, UBB, and UBC^4^. Deubiquitylases (DUBs) play a key role in generating free Ub from these precursors^5^. Ub can be attached covalently to lysine (K) residue/s of substrate proteins to determine their fate in a process called ubiquitylation. Alternatively, K residues can also be covalently attached by Ub-like modifiers such as small ubiquitin-like modifier (SUMO)^6^ or neuronal precursor cell-expressed, developmentally down-regulated protein 8 (NEDD8)^7^, which are structurally and biochemically highly similar to Ub and they also depend on the sequential action of dedicated activating, conjugating, and ligating enzymes as described below for Ub^8^.

**Fig. 1:**
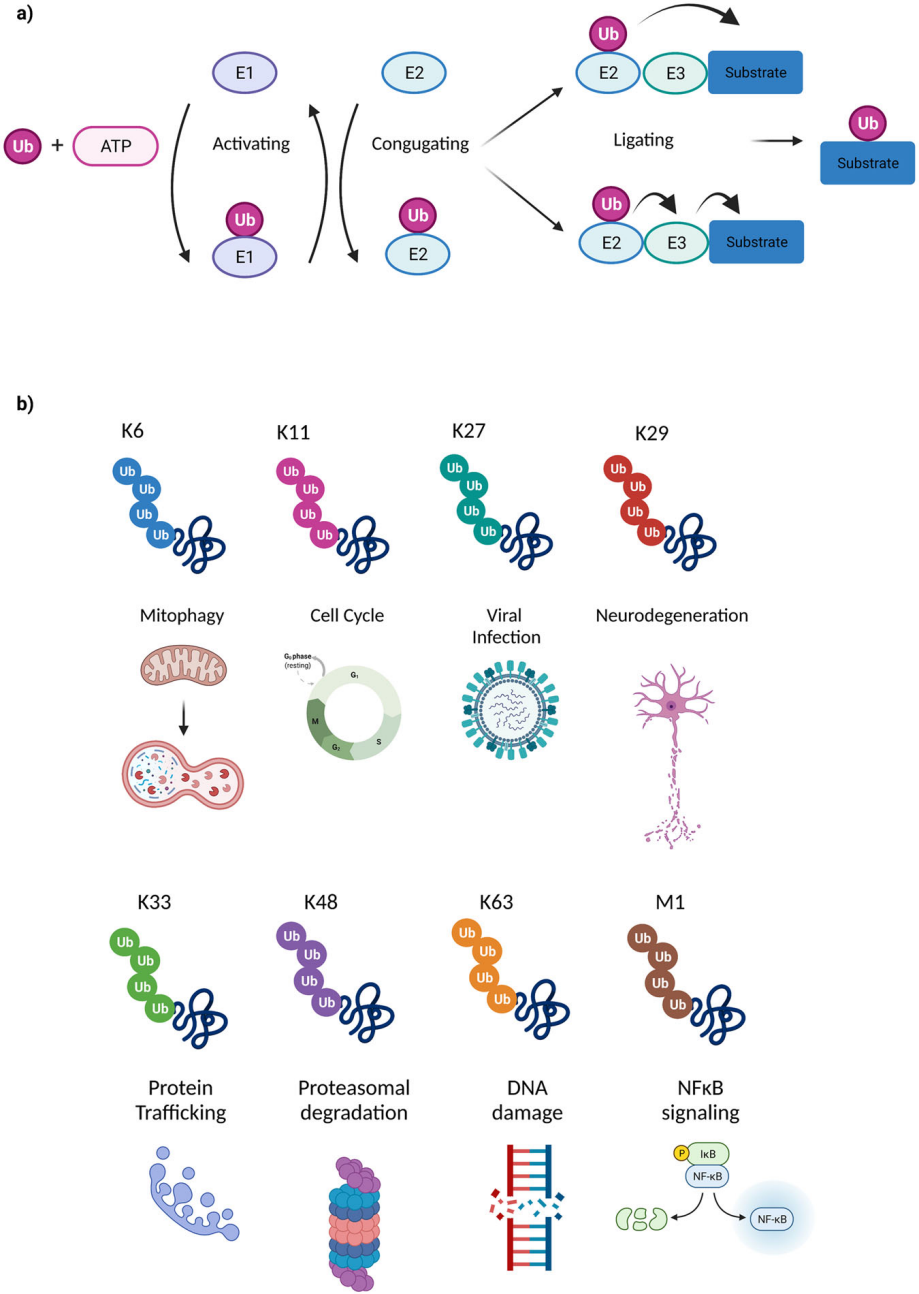
The ubiquitin code in a snapshot. **a** Ubiquitylation is an ATP-dependent process that depends on the cooperative action of three sequential enzymes: Ub-activating enzyme (E1), Ub-conjugating enzyme (E2), and Ub E3 ligase (E3). Ub (magenta) is attached covalently to a lysine (K) residue of a substrate protein (dark blue) either through direct transfer from the E2 enzyme to the substrate, or through sequential transfer from the E2 enzyme to the E3 ligase and then to the substrate. **b** Ubiquitin can form eight different linkage types, using one of seven internal lysine residues (K6, K11, K27, K29, K33, K48, K63) or methionine at position 1 (M1). Specific Ub chains are capable to confer distinct functions of targeted substrates and thereby regulate different cellular processes. The functions illustrated here for each given type of Ub-chain are not restricted to the biological pathway depicted, but are rather representative examples chosen for simplicit.

The C-terminal glycine of Ub is typically bound to K residue/s of substrate protein through the cooperative action of three enzymes: Ub-activating enzyme (E1), Ub-conjugating enzyme (E2), and Ub ligase (E3)^9^ (Fig. 1a). First, the C-terminal carboxyl group of Ub is activated by E1, coupling ATP hydrolysis to the formation of a high-energy thioester bond with the catalytic cysteine (C) residue of E1. Subsequently, Ub is delivered to the catalytic C residue of E2 in a process termed E1–E2 thioester transfer. Finally, E3 ligase selectively recognizes both the E2 and the substrate, and catalyzes Ub transfer from the E2 to the K residue/s of the target protein through the formation of an isopeptide bond^10,11^. In addition to determining substrate specificity, E3 ligases interact with auxiliary factors in order to limit the E2 conjugation potential and direct the attachment of polyubiquitin (polyUb) to the desired lysine residue on the target substrate^12^. Similar to phosphorylation and dephosphorylation, ubiquitylation and deubiquitylation are dynamic processes where specialized DUBs can remove Ub chains from target proteins opposing the function of E3s^13^.

Human E3 ligases can be classified into three main categories: really interesting new gene (RING) which counts more than 600 members, homologous to E6-AP carboxyl terminus (HECT) which has ~28 members and ring-between-ring (RBR) which includes 14 members^14^. There are only two known E1 enzymes, around 40 E2 proteins, and ~100 DUBs in human cells^15–17^. Ubiquitylation is a versatile PTM involved in many cellular processes including: cell proliferation, DNA repair, transcriptional regulation, viral infection, immune response, apoptosis, angiogenesis, and metastasis^18–20^. This diversity originates from the fact that Ub protein itself can also be modified by additional Ub molecules^21^. Ub contains seven K residues (K6-, K11-, K27-, K29-, K33-, K48-, and K63-) and all of them can be ubiquitylated, giving rise to various isopeptide-linked Ub chains^22^ (Fig. 1b). Additionally, α-amino group on the N-terminal methionine 1 (M1) of Ub can also be ubiquitylated, creating the eighth chain type, the M1-linked (or linear) polyUb chain. The wide variety of possible Ub modifications can be referred to as the Ub code and include monoubiquitylation, multi-monoubiquitylation, homotypic polyubiquitylation (through the same Ub K residue), and heterotypic polyubiquitylation (mixed, branched, SUMO- or NEDD8-modified or even phosphorylation- or acetylation-modified chains)^23,24^.

It is remarkable that specific Ub chains are capable to confer distinct functions on targeted substrates and thereby regulate different cellular processes (Fig. 1b). Although the general trend is that K48-linked polyUb chains target modified proteins to the 26S proteasome for their degradation^25–27^, some additional K-linked chains can accumulate upon proteasomal inhibition, suggesting that other Ub chains share similar function and contribute to targeted protein destruction^28^. However, increasing evidence for non-proteolytic functions mostly mediated by non-K48-linked ubiquitin chains has been widely documented. M1-linked and K63-linked chains are broadly covered in the literature, with M1 chains playing an important role in cellular death, immune response, and protein quality control^29,30^, and K63 chains being important for endocytic trafficking, inflammation, and DNA repair^31–33^. On the other hand, other types of Ub linkages are still poorly characterized regarding their cellular functions, with a recent review shedding light onto the respective individual signaling cascades^34^. Briefly, K6 is involved in mitophagy^35,36^ or protein stabilization^37–39^, K11 have roles in DNA damage response (DDR)^40^, K27 play roles in innate immunity^41^, K29 are implicated in the Wnt/β-catenin signaling and in neurodegenerative disorders^42,43^ and finally K33 regulate protein trafficking^44^. Figure 1b provides an overview of the above-mentioned examples, however, it is important to note that the functions of one given type of Ub-chain are not restricted to a particular biological pathway, especially considering the fact that very often chains are branched and therefore use a mixture of Ub-linkages.

Intriguingly, the prevailing dogma that lysine is the canonical site for substrate ubiquitylation has been heavily challenged in the last years, with cysteine (C), serine (S), and threonine (T) residues emerging as functional ubiquitylation sites through the formation of thioester and hydroxyester bonds^45^. C-dependent ubiquitylation was first described to regulate virus-induced endocytocis^46^ and later it was linked to peroxisome import receptor signaling^47,48^, whereas S/T-dependent ubiquitylation is mostly studied in the context of endoplasmic-reticulum-associated protein degradation (ERAD)^49,50^. Furthermore, a unique mechanism of phosphoribose-linked ubiquitylation conjugated to serine residues catalyzed by the SidE effector proteins has evolved in the *Legionella* bacteria family, which is extensively studied in the context of reprogramming the host cell during bacterial infection and in regulating organelle dynamics^51,52^.

In this review, we discuss the latest concepts on how non-proteolytic ubiquitylation pathways are involved in various physiological and pathological conditions, such as cell division, organelle dynamics, development, and cancer. Table 1 summarizes how non-proteolytic ubiquitylation is implicated in cellular signaling and Table 2 outlines the involvement of non-proteolytic ubiquitylation in disease.

**Table 1:** List of non-proteolytic ubiquitylation events described in different cellular signaling pathways.

E2/E3/DUB	Substrate	Ub Linkage	Phenotype	Reference
DNA Damage Response (DDR)				
RNF168 (E3)	H2A/H2A.X	K27	Promotes recruitment of DDR proteins to DNA damage foci	^56^
UBC13 (E2) RNF8 (E3)	H1	K63	Promotes RNF168 recruitment to DSBs sites	^57^
RNF8 (E3)	Akt	K63	Promotes the translocation of Akt to the plasma membrane or Akt binding to DNA-PKcs	^58^
SPOP (E3)	Geminin	K27	Prevents DNA replication over-firing	^60^
SPOP (E3)	53BP1	K29	Reduces 53BP1 recruitment to DSBs sites and promotes DNA repair by the HR pathway	^61^
UCHL3 (DUB)	RAD51	?	Promotes Homologous Recombination (HR) repair pathway	^62^
Cell Division				
CUL3/KLHL22 (E3)	PLK1	mono	Removes PLK1 from the kinetochores to ensure the timely initiation of anaphase	^66–68^
CUL3/KLHL9/13/21 (E3)	AuroraB	mono	Removes Aurora B from the centromeres to ensure the timely initiation of anaphase	^70–73^
UCHL3 (DUB)	AuroraB	?	Promotes chromosome bi-orientation and faithful chromosome segregation	^78^
CUL4A^RBX1-COPS8^ (E3)	CENP-A	?	Promotes CENP-A recruitment to the centromere and is required for proper CENP-A inheritance	^79–83^
MGRN1 (E3)	α-tubulin	K6	Promotes α-tubulin polymerization and microtubule-based transport	^37, 38^
TRIM37 (E3)	PLK4	?	Inhibits PLK4 self-assembly into ectopic microtubule-nucleating centers and ensures mitotic fidelity	^87, 88^
CUL1, CUL3, CUL4A and CUL4B	PLK1, TBK1, INCENP and MLKP1	K29	Regulates midbody assembly	^89^
Organelle Dynamics-Transport				
UBE2J1 (E2) RNF26 (E3) USP15 (DUB)	SQSTM1 (p62)	?	Regulates vesicle maturation and cargo trafficking	^90, 91^
CUL3/KLHL12 (E3)	Lunapark	?	Regulates translocation and activation of mTORC1 to the lysosome	^92^
USP32 (DUB)	Rab7	?	Promotes Rab7 recycling and transport activity	^93^
Parkin (E3)	Rab7	?	Increases Rab7 activity and regulates exosome secretion	^94^
^Development^				
RNF220 (E3)	GliA and GliR	K63	Controls GliA and GliR nucleocytoplasmic shuttling and activation gradient during neural patterning	^97^
MKRN3 (E3)	MDB3	K27	Promotes epigenetic silencing of *GNRH1* transcription	^98^
MKRN3 (E3)	PABPC1	K27, K29	Inhibits formation of the translation initiation complex	^99^
WWP2 (E3)	RUNX2	?	Promotes transcriptional and osteogenic activity of RUNX2	^101^
SCF^SKP2^ (E3) OTUD1 (DUB)	YAP	K63	Fine-tunes YAP localization, transcriptional activity and function	^103^
CUL3/KCTD10 (E3)	EPS8-IRSp53	?	Controls the remodeling of actin cytoskeleton and promotes cell fusion	^105^
NF-κB pathway				
TRIM32 (E3)	OTULIN	K63	Promotes NF-κB activation downstream of LUBAC	^109^
USP19 (DUB)	TAK1	K27, K63	Attenuates inflammatory response	^111^
cIAP1/cIAP2 (E3)	BCL10	K63	Promotes the recruitment and activation of LUBAC/IKK/NEMO signaling upstream of NF-κΒ	^112^

**Table 2:** List of non-proteolytic ubiquitylation events described in different disease-mediating processes.

E2/E3/DUB	Substrate	Ub Linkage	Phenotype	Reference
Cancer				
SMURF1 (E3) ZRANB1 (DUB)	UVRAG	K29, K33	Promotes autophagosome maturation and inhibits HCC tumor growth	^115^
ITCH^β-Arrestin2^ (E3)	SuFu	K63	Increases SuFu binding to Gli transcription factors and controls medulloblastoma tumorigenesis downstream of Hedgehog signaling	^116^
ITCH (E3)	BRAF	K27	Promotes melanoma proliferation and invasion downstream of ERK signaling	^117^
CUL4^AMBRA1^	SMAD4	K63	Promotes EMT, migration, invasion and metastasis of breast cancer cells downstream of TGF-β signaling	^119^
RNF181 (E3)	YAP	K48	Promotes Triple Negative Breast Cancer cell proliferation, migration and invasion downstream of Hippo signaling	^120^
UBE2S (E2)	β-Catenin	K11	a) Promotes differentiation of embryonic stem cells into mesoendoderm lineages b) Promotes colorectal cancer cell proliferation and metastasis	^121^
TRAF4 (E3)	TRKA	K27, K29	Promotes prostate cancer cell migration, invasion and metastasis	^122^
CBLC (E3)	EGFR	K6, K11	Inhibits EGFR lysosomal degradation and promotes lung cancer cell viability	^39, 123^
Metabolism-related diseases				
SMURF1 (E3)	PPARγ	K63	Suppresses PPARγ transcriptional activity and inhibits lipid accumulation in the liver, protecting from NAFLD	^125^
UBE2N/UBC13 (E2)	Akt	K63	Regulates insulin sensitivity in a GPS2-dependent manner to control lipid accumulation and obesity	^133^
HECTH9/HUWE1 (E3)	HK2	K63	Promotes glucose metabolism, cancer stem cell (CSC) expansion and CSC-associated chemoresistance	^138^
Parkin (E3) OTUB2 (DUB)	PKM2	?	Regulates PKM2 enzymatic activity and glycolysis	^139, 140^
Innate Immunity-related diseases				
OTUD1 (DUB)	IRF3	K6, K63	Attenuates inflammatory response after HSV-1 and VSV infection	^142, 143^
HECTD3 (E3)	MALT1, STAT3	K27, K29	Promotes differentiation of Th17 cells downstream of NF-kB activation	^147^
SCF^Skp1-Cullin1-Fbxo21^ (E3)	ASK1	K29	Promotes type I interferon production upon VSV and HSV-1infection	^149^
CUL4^DCAF12L1/ DCAF11^ (E3)	PB2	K29	Promotes Influenza A Virus (IAV) infection	^150^
TRIM21 (E3)	MAVS	K27	Activates innate immune response and inhibits RNA viral infection	^151^

## Non-proteolytic ubiquitylation in the DNA damage response (DDR)

**Fig. 2:**
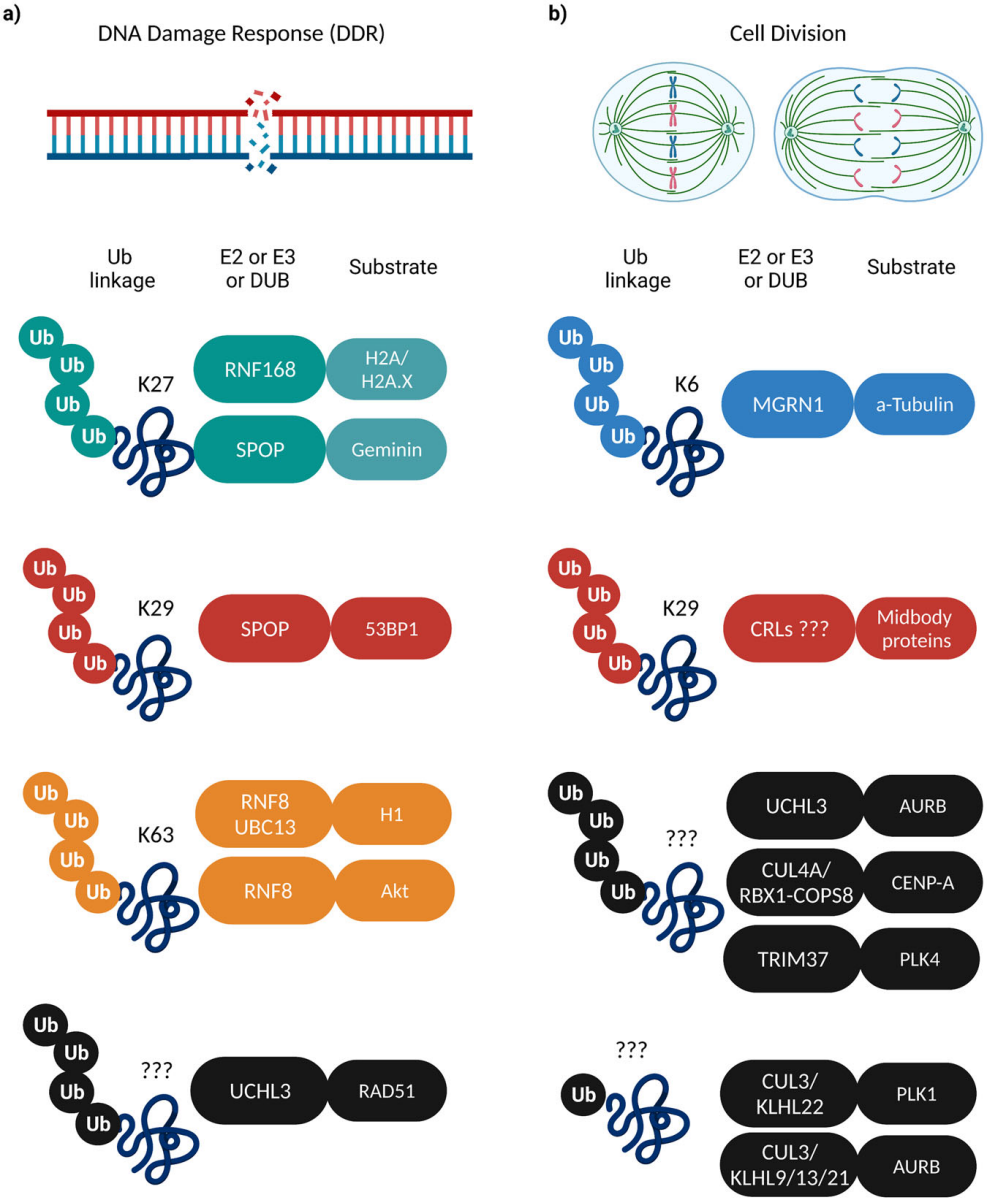
Non-proteolytic ubiquitylation in DDR and cell division signaling. Examples of reported E2/E3/DUB enzymes (left boxes) and different non-proteolytic Ub signals on the substrate proteins (right boxes) regulating **a** DDR and **b** cell division signaling pathways.

The DDR is a surveillance network of cellular pathways that can sense DNA damage and initiate diverse signaling cascades which will determine the choice of repair pathway between non-homologous end-joining and homologous recombination (HR), in order to preserve genome integrity^53^. Histone ubiquitylation and K63-Ub linkages are major determinants of DDR and have been extensively reviewed for their non-proteolytic ubiquitin functions, with the prevailing dogma suggesting that they serve as recruitment platforms for downstream repair effectors at the DNA damage sites^54,55^. Interestingly, accumulating evidence suggests that DDR can also be stimulated by non-K63 chains. In response to genotoxic stress, the RING E3 ligase RNF168 marks the chromatin histones H2A and H2A.X through K27 ubiquitylation^56^ (Fig. 2a). This modification is essential for the generation of docking sites and the subsequent recruitment of established DDR modulators including TP53-binding protein 1 (53BP1) and breast cancer type 1 susceptibility protein (BRCA1) to DNA damage sites, suggesting an additional level of complexity of the histone ubiquitin code in DDR that warrants further investigation. While RNF168 is known to catalyze the ubiquitylation of the core H2A-type histones, it has been demonstrated that another E3 ligase is responsible for modifying the H1-type linker histones upon DNA double-strand breaks (DSBs) formation. The complex consisting of RNF8 E3 ligase and UBC13 E2 conjugating enzyme, mediates the K63-linked ubiquitylation of H1 histones and provides an initial binding platform that triggers RNF168 recruitment, a step which is required for the optimal amplification of the ubiquitin signaling after DNA damage induction^57^ (Fig. 2a). RNF8 was recently suggested to act as a critical modulator of Akt kinase activation through direct K63-linked ubiquitylation both under physiological and genotoxic conditions^58^ (Fig. 2a). More specifically, the authors show that upon growth factors stimulation, RNF8 promotes the translocation of Akt to the plasma membrane, while upon conditions of DNA damage induction RNF8 facilitates the binding of Akt to DNA-PKcs (DNA-dependent protein kinase catalytic subunit). Both signaling cascades are known to be a prerequisite for the phosphorylation-dependent activation of Akt, underscoring an important role for RNF8-mediated ubiquitylation in driving Akt hyperactivation, which can be correlated with enhanced cancer cell survival.

Speckle-type BTB/POZ protein (SPOP) is a substrate recognition receptor of the cullin-3 (CUL3)/RING-type ubiquitin E3 complex that has emerged as a gatekeeper of genome stability given its critical roles in DDR and DNA replication^59^. Recently, two studies further extended the role of SPOP in maintaining DNA integrity through non-proteolytic ubiquitylation and identified 53BP1 and Geminin as substrates of the CUL3/SPOP complex^60,61^ (Fig. 2a). SPOP promotes the K27-linked non-degradative poly-ubiquitylation of the essential DNA replication protein Geminin during S phase and prevents DNA replication over-firing by inhibiting the interaction of the Geminin binding partner Cdt1 with the MCM (mini-chromosome maintenance) complex^60^. Cancer-associated SPOP mutations impair Geminin K27-linked poly-ubiquitylation and lead to replication stress and cell death upon ATR kinase inhibition, implying that cancers with defective SPOP-Geminin signaling might be responsive to ATR inhibitors treatment. Moreover, SPOP interacts with 53BP1 primarily during S phase and catalyzes its K29-linked polyubiquitylation, which triggers its exclusion from chromatin and reduces its presence at DSB sites by enhancing 53BP1 interaction with the valosin-containing protein/p97 cofactor NPL4^61^. This ubiquitylation is enriched in response to DNA damage, where ATM kinase phosphorylates SPOP in order to induce a conformational change in SPOP that stabilizes its interaction with 53BP1. The authors suggest that SPOP functions as a factor that promotes DNA repair by the HR pathway in the context of chromatin-engaged 53BP1, and that SPOP mutations frequently found in cancer patients are responsible for inducing genomic instability due to attenuated SPOP-53BP1 interaction that results in defective HR. Finally, the Ubiquitin C-terminal Hydrolase L3 (UCHL3), a DUB mostly known for its function in the processing of Ub precursors and in cell division, was recently identified as an unexpected hit in a shRNA based screening for DUBs with roles in DNA repair^62^. Following DNA damage, UCHL3 deubiquitylates RAD51, a critical component of the HR pathway, facilitating its binding to BRCA2 and consequently its recruitment to DBSs sites (Fig. 2a). Of note, elevated UCHL3 expression in breast cancer cells was shown to render them resistant to radiation and chemotherapy due to increased ability for DNA repair in a RAD51-dependent manner, suggesting that this new signaling axis could be exploited therapeutically in anticancer treatments.

## Non-proteolytic ubiquitylation in cell division

Ubiquitylation is also critically involved in the regulation of cell division. The E3 ligase anaphase-promoting complex/cyclosome (APC/C) is the main enzyme targeting essential mitotic factors for proteasomal degradation. These include securin and cyclin B which critically regulate sister-chromatid separation and mitotic exit, respectively^63^. However, increasing evidence suggests that also non-proteolytic ubiquitylation plays an important role during mitotic progression^64^. Our team has extensively studied the non-proteolytic ubiquitylation pathways mediated by the RING-based CUL3 E3 ligases in mitosis^65^. CUL3 engages the adapter protein KLHL22 to regulate dynamic localization of Polo-like kinase 1 (PLK1) to kinetochores^66–68^. CUL3/KLHL22 monoubiquitylates K492 of PLK1, leading to the dissociation of PLK1 from the kinetochores and ensuring timely metaphase to anaphase transition (Fig. 2b). The absence of K492-ubiquitylation increases the binding of PLK1 to kinetochore-associated phosphoreceptors, resulting in prolonged mitotic arrest and cell death. Since PLK1 represents a mitotic factor with strong clinical relevance in cancer^69^, modulating specifically the kinetochore-associated pool of PLK1 could represent an interesting strategy to induce cell death in mitotic cancer cells.

CUL3-based complexes can also monoubiquitylate K56 on Aurora B kinase, regulating its localization to chromosomal arms and microtubules^70–73^. Deletion of CUL3 or of Ub receptor Ubiquitin-associated (UBA) and SH3 domain-containing protein B (UBASH3B), leads to defects in the centromeric focusing of Aurora B and inhibits its relocalization to microtubules, leading to chromosome alignment and segregation errors, cytokinesis defects, and cell death^70–73^ (Fig. 2b). It is interesting to note that UBASH3B is overexpressed in several cancers and its oncogenic potential has been demonstrated in the context of leukemia and breast cancer^74,75^, with at least some of these effects being related to the mitotic function of UBASH3B^76^. Thus, CUL3-based E3 ligases can act as both oncogenes and tumor suppressors depending on the binding to specific substrate-specific adapter proteins and on the cellular/tissue context^77^. Although the identity of the DUBs specifically opposing CUL3-mediated monoubiquitylation events during mitosis is currently unknown, we recently showed that UCHL3 regulates non-proteolytic polyubiquitylation of Aurora B^78^ (Fig. 2b). UCHL3 promotes the kinetochore localization of Aurora B and monitors bi-orientation of chromosomes, adding another level of complexity to the regulation of Aurora B for faithful chromosome segregation.

Several lines of research from the Kitigawa lab have focused on how the centromere-specific histone H3 variant (CENP-A) determines centromere identity, inheritance, and function in order to ensure the fidelity of the genome, and have highlighted the indispensable role of non-proteolytic ubiquitylation of CENP-A in regulating its deposition at the centromere (Fig. 2b). The CUL4A-RBX1-COPS8 (Cullin 4A (CUL4A)—RING-box protein 1 (RBX1)—COP9 signalosome complex subunit 8 (COPS8) complex ubiquitylates CENP-A on K124 and this modification is critical for CENP-A interaction with the chromatin assembly factor Holliday junction recognition protein (HJURP) and for CENP-A localization to centromeres^79^. Of note, the CUL4A–RBX1–COPS8 complex is not only required for incorporating newly synthesized CENP-A into centromeres, but also for maintaining there the old CENP-A pools at this cellular structure. The latter observation was further corroborated by the observation that preexisting ubiquitylated CENP-A is a prerequisite for the recruitment of newly synthesized CENP-A to the centromere and that CENP-A ubiquitylation is inherited during following cell divisions^80^. The authors demonstrate that both forms of CENP-A (old and new) are ubiquitylated before S phase and that after DNA replication HJURP preferentially binds to ubiquitylated, preassembled old CENP-A, which then triggers the recruitment of new CENP-A to the centromere. The formation of this CENP-A heterodimer (old and new dimers) is essential for the recognition of CENP-A by the CUL4 E3 ligase which will subsequently lead to the ubiquitylation of new CENP-A. These results suggest that the ubiquitylation and the position of the centromere are inherited epigenetically, with CENP-A ubiquitylation serving as a memory event preventing the formation of centromeres at other places on chromosomes, thus rendering the CUL4-mediated ubiquitylation of CENP-A indispensable for cell viability^81,82^. The importance of CUL4-mediated non-proteolytic ubiquitylation of CENP-A in maintaining centromere identity and function was heavily disputed by a study showing that ubiquitylation of CENP-A at K124 is required only during the early steps of centromere establishment, whereas it is dispensable for the long-term centromere maintenance and for the centromeric chromatin assembly in G1 phase^83^. Contrary to previous studies, the ubiquitylation status of CENP-A did not have any effect on its interaction with HJURP or on its centromeric deposition, suggesting that this modification is not involved in generating or maintaining functional centromeric chromatin. Considering that impairment of centromere integrity is highly associated with chromosome instability and aneuploidy^84^, further investigation should be warranted to clarify the discrepancy regarding the role of CUL4-mediated non-proteolytic ubiquitylation of CENP-A in regulating centromere inheritance and function.

During mitosis, the movement and segregation of duplicated chromosomes are orchestrated by the mitotic spindle, a bipolar structure built by microtubules comprising of α/β-tubulin heterodimers. Recently, the emerging concept of the tubulin code is suggested to add an extra layer of complexity in terms of microtubule function and mitotic progression, that largely relies on a variety of PTMs on tubulin isoforms, including non-proteolytic ubiquitylation^85,86^. The E3 ligase Mahogunin ring finger-1 (MGRN1) modifies specifically α-tubulin via K6-linked polyubiquitylation and promotes its polymerization, leading to mitotic spindle misorientation^37^ (Fig. 2b). The authors further demonstrate that MGRN1-mediated α-tubulin ubiquitylation is critical for microtubule-based transport and that the intracellular transport of mitochondria and endosomes is impaired when this ubiquitylation is compromised^38^.

Recent evidence identified the tripartite motif E3 ligase TRIM37 as an essential player in ensuring mitotic fidelity via the non-proteolytic ubiquitylation of centrosome-related substrates specifically under conditions of acentrosomal microtubule spindle assembly^87,88^. The authors demonstrate that TRIM37 ubiquitylates PLK4 (polo-like kinase 4) in order to inhibit PLK4 self-assembly into condensates which act as a scaffold for the recruitment of additional centrosomal components and which eventually function as ectopic microtubule-nucleating centers to promote acentrosomal division^87^ (Fig. 2b). The ability of TRIM37 to prevent the formation of ectopic PLK4-containing aggregates is particularly important in the context of cells lacking functional centrosomes, suggesting for example that TRIM37 could trigger selective mitotic failure in cancer types sensitive to PLK4 inhibition.

While the function of K29 linkages is still obscure compared to other Ub chain topologies, a novel study revealed their interaction landscape^89^. The authors show that K29-ubiquitylation emerges as an important player in cell cycle regulation and progression and that this modification is particularly enriched at the midbody during telophase. More specifically, K29 ubiquitin chains strongly interact and modify crucial components required for midbody assembly such as PLK1, TAK-binding kinase 1 (TBK1), INCENP, and MLKP1 in a non-degradative fashion (Fig. 2b). In addition, the K29 midbody fractions do not co-localize with 20S proteasome or K48 chains, but rather with VCP/p97 (also called CDC48 in certain eukaryotes), further supporting a role for K29 in midbody assembly that needs to be investigated in more detail.

## Non-proteolytic ubiquitylation in the regulation of organelle homeostasis

The role of ubiquitin in regulating organelles dynamics and homeostasis has been mostly studied in the context of ERAD. However, how the ubiquitin system is functionally intertwined with retro-translocation machinery to transport polypeptides across the ER membrane remains poorly understood. Non-proteolytic ubiquitylation has emerged as a critical determinant of the spatiotemporal regulation of vesicle trafficking between the ER and the endosome. Studies from the Neefjes group have elucidated the major components of an ER-associated ubiquitin network that shapes the endosomal architecture as a means to orchestrate vesicle maturation and cargo trafficking in space and time^90,91^. The E3 ubiquitin ligase RING finger protein 26 (RNF26) localizes at the ER where it serves as a platform for the perinuclear positioning of the entire endosomal system through direct ubiquitylation of the p62/sequestosome 1 (SQSTM1) scaffold protein, which in turn recruits various vesicle membrane adapters^90^. As a result, vesicles are trapped by this multi-protein complex and are forced to be retained in the perinuclear space, until the counteracting action of the USP15 DUB on SQSTM1 allows the targeted vesicles to regain mobility and to be released for fast microtubule-based transport towards the cell periphery. Later on, UBE2J1 was identified as the E2 conjugating enzyme that interacts with RNF26/SQSTM1 in order to regulate the RNF26-mediated anchoring of vesicle adapters and the perinuclear organization of the endolysosomal system^91^, thus further demonstrating the importance of non-proteolytic ubiquitylation in the spatiotemporal control of organelle dynamics with possible implications in membrane-dependent signaling pathways.

Within the same context of organelle compartmentalization and intracellular transport, a proteomic screen searching for Cullin-RING E3 ligase (CRL) substrates that are specifically ubiquitylated at cellular membranes, identified the ER-shaping protein Lunapark as a non-proteolytic target for the ubiquitin ligase CUL3/KLHL12^92^. Interestingly, Lunapark ubiquitylation is not required for its ER localization and does not regulate ER architecture or dynamics, but is indispensable for the translocation and activation of mTORC1 (mechanistic target of rapamycin complex-1) at the lysosome. Non-ubiquitylated Lunapark fails to interact with mTOR at ER-lysosome contact sites, leading to hyperactivation of downstream growth signaling pathways in response to nutrients. Moreover, zebrafish embryos defective for CUL3/KLHL12- mediated Lunapark ubiquitylation displayed increased locomotion activity and neurodevelopmental defects, suggesting that future investigation on how Lunapark ubiquitylation might be implicated in neurodegenerative diseases associated with mTOR hyperactivation should be warranted. Another screen identified DUB USP32 as a critical modulator of endosomal membrane dynamics and architecture by directly deubiquitylating the small GTPase Rab7 at K191 and promoting its functions in transport and recycling from the multi-vesicular body via two distinct mechanisms^93^. Deubiquitylation of Rab7 by USP32 promotes its release from the membrane to the cytosol and its binding to the late endosome (LE) transport effector Rab-interacting lysosomal protein (RILP), thereby favoring LE transport toward the nucleus. Moreover, in the presence of USP32, Rab7 association with the retromer (a complex of proteins important in recycling transmembrane receptors from endosomes to the trans-Golgi network) is destabilized and leads to enhanced membrane recycling from the LE. According to another study related to endosomal organization and function, Rab7 is also subjected to non-proteolytic ubiquitylation by the E3 ligase Parkin^94^. Parkin ubiquitylates Rab7 at K38 and increases its activity, its binding to the RILP effector and its membrane association, and is suggested to at least partially be responsible for reduced exosome secretion. Considering that all above-mentioned components (USP32, Parkin, Rab7), as well as deregulation of endosomal membrane dynamics have been implicated in the pathogenesis of Parkinson’s disease (PD), we could speculate that non-proteolytic ubiquitylation signaling within the endolysosomal system might provide new perspectives into the development of therapeutic strategies against PD.

## Non-proteolytic ubiquitylation and development

Components of the ubiquitin machinery have been shown to control different aspects of embryonic development by modulating a variety of developmental signaling pathways. Here, we outline some recent examples of how non-proteolytic ubiquitylation directs developmental cell-fate decisions.

**Fig. 3:**
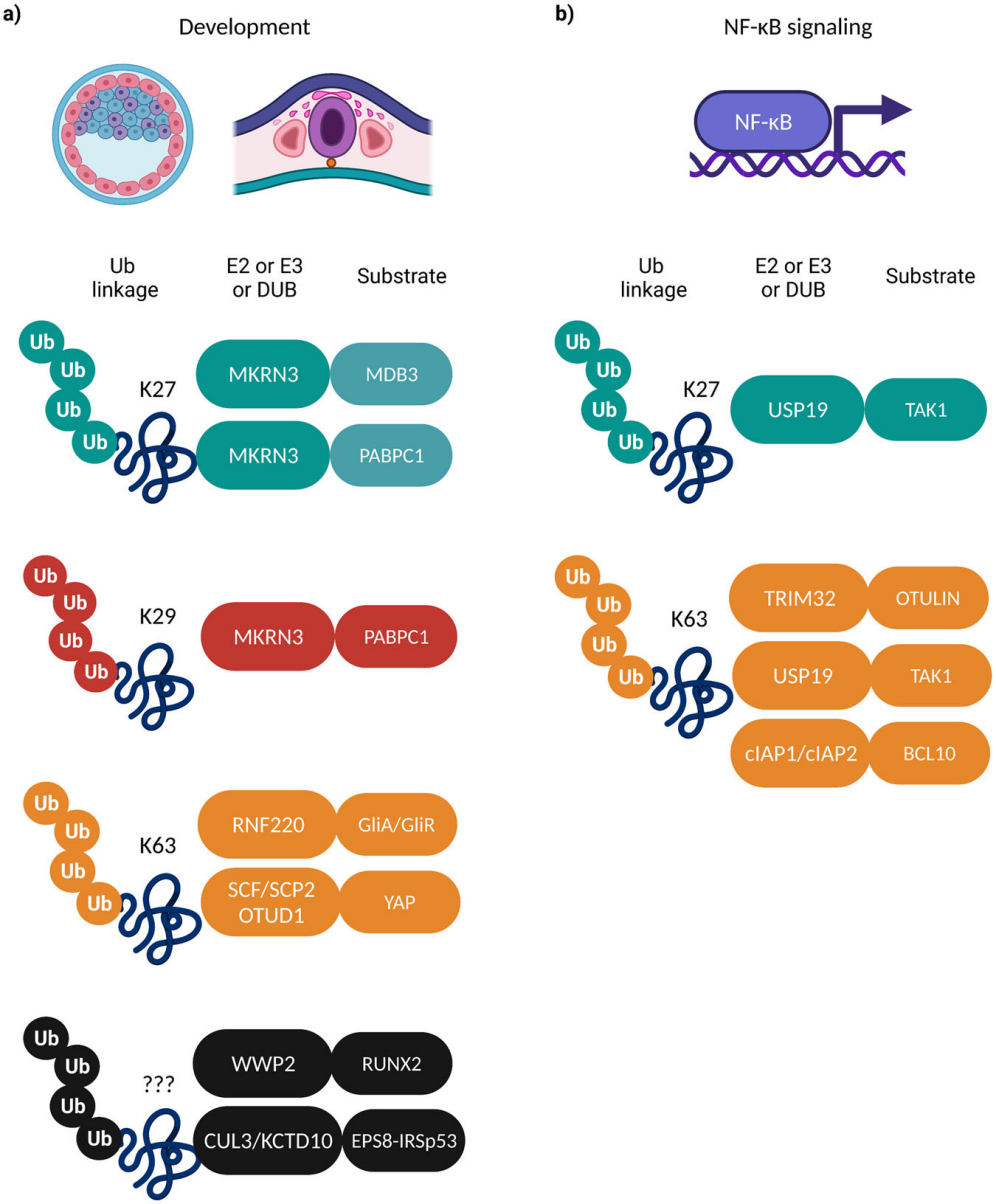
Non-proteolytic ubiquitylation in developmental processes and NF-κB signaling. Examples of reported E2/E3/DUB enzymes (left boxes) and different non-proteolytic Ub signals on the substrate proteins (right boxes) regulating **a** development and **b** NF-κB signaling pathways.

Neural patterning is the biological process by which cells in the developing nervous system acquire distinct identities according to their specific spatial positions^95^. One of its major regulators is the Sonic Hedgehog signaling which fine-tunes the balance of Gli activators (GliA) and repressors (GliR) in the ventral neural tube to govern the local expression of a group of cell-fate-determining transcription factors in responding neural progenitors^96^. A recent study addressed the role of non-proteolytic ubiquitylation in regulating the Gli gradient and identified the RING E3 ligase RNF220 as a novel important modulator of neural patterning in mice embryos^97^ (Fig. 3a). RNF220 interacts and ubiquitylates both activators and repressors of the Gli family and controls their nucleocytoplasmic shuttling by unmasking a nuclear export signal that is then recognized by the exportin CRM1. Disruption of this ubiquitylation leads to enhanced Gli activation in the nucleus and subsequently interferes with the GliA and GliR gradient equilibrium, thereby resulting in irregular neural cell differentiation patterns.

Studies from the Hu lab have identified the RING-type E3 ligase MKRN3 (Makorin 3) as a critical repressor of premature mammalian puberty initiation through the non-proteolytic ubiquitylation of several substrates within the gonadotropin-releasing hormone (GnRH) signaling pathway at both translational and epigenetic level^98,99^ (Fig. 3a). MKRN3 ubiquitylates methyl-DNA binding protein 3 (MBD3) in order to inhibit both its binding to the promoter of *GNRH1* and its interaction with the demethylase TET2 (ten-eleven translocation methylcytosine dioxygenase 2), hence leading to the epigenetic silencing of *GNRH1* transcription^98^. Moreover, MKRN3 ubiquitylates (Poly (A)-binding protein 1(PABC1)) in order to decrease its binding to the poly (A) tails of *GNRH1* mRNA, which results in *GNRH1* mRNA destabilization and disruption of the translation initiation complex formation^99^. Both studies have provided important genetic evidence for the role of MKRN3-dependent non-proteolytic ubiquitylation in the inhibition of the early activation of hypothalamic axis upstream of puberty initiation and development, suggesting new targets for the treatment of clinical conditions like central precocious puberty.

Bone remodeling is a critical process of the adult skeleton homeostasis that depends on the balanced actions of osteoblasts and osteoclasts. Among the master regulators that are involved in the commitment of mesenchymal progenitors to differentiate into osteoblasts, are the bone morphogenetic proteins (BMPs), the RUNX Family Transcription Factor 2 (RUNX2), and the proteolytic signaling mediated by the NEDD4 family of HECT type E3 ligases^100^. A recent study provides evidence for the involvement of the WWP2 E3 ligase in osteogenic differentiation by catalyzing the non-proteolytic mono-ubiquitylation of RUNX2 in order to stimulate its transcriptional and osteoblastic actitivity, a modification that is shown to be further enhanced upon BMP signaling activation^101^ (Fig. 3a). Considering that missense mutations linked to severe skeletal disorders have been identified for the WWP2 targeted lysine residues of RUNX2, it would be important to further dissect the role of non-proteolytic ubiquitylation events in osteogenesis and skeleton development.

Yes-associated protein (YAP) is a transcription factor with established roles in development and in oncogenic processes. YAP continuously shuttles between the nucleus and the cytoplasm and the regulation of its activity has mostly been studied downstream of the Hippo pathway^102^. A Hippo independent mechanism based on the non-proteolytic ubiquitylation of YAP was recently described to finetune its localization and function through the counteracting actions of the SCF^SKP2^ E3 ligase complex and the DUB OTUD1^103^ (Fig. 3a). SKP2 ubiquitylation enhances the interaction of YAP with its nuclear binding partner TEAD and induces YAP nuclear localization, transcriptional activity, and growth-promoting function, while OTUD1-mediated deubiquitylation promotes the retention of YAP in the cytoplasm and blocks its activity.

Dynamic remodeling of the actin cytoskeleton is a key process driving intracellular communication and fusion during tissue formation that needs to be precisely coordinated to prevent developmental defects^104^. Non-proteolytic monoubiquitylation mediated by the CUL3/KCTD10 complex was recently identified as a molecular rheostat that finetunes actin bundling in a model of myogenic cell fusion by exerting dual counteracting activities on the EPS8–IRSp53 complex^105^ (Fig. 3a). The authors demonstrate that CUL3/KCTD10-dependent ubiquitylation of EPS8-IRSp53 is regulating both the recruitment as well as the displacement of the complex to the plasma membrane in order to timely orchestrate the actin-bundling at cell–cell contact sites to promote myoblast fusion. This mechanism was further shown to not only be required for myoblast-myotube fusion, but rather to be responsible for generally rearranging the actin network and restricting actin-bundling when it is no longer required, opening research avenues that exploit this signaling in pathological conditions characterized by defective cell fusion.

## Non-proteolytic ubiquitylation in NF-κB signaling

Nuclear factor kappa light chain enhancer of activated B cells (NF-κB) is a widely studied family of transcription factors, highly involved in various processes such as inflammation, immune response, and cell survival. The multiple biological mechanisms that mediate NF-κB signaling, as well as the genetic diseases that are associated with deregulated NF-κB pathways have been extensively reviewed^106^. Mounting evidence demonstrates that non-proteolytic ubiquitylation is one of the major PTMs that control the NF-κB cellular response, with the M1-linked ubiquitylation mediated by the RBR-type E3 ligase linear ubiquitin chain assembly complex (LUBAC) remaining in the spotlight^107^. Furthermore, the deubiquitylase OTULIN (OTU domain-containing deubiquitinase with linear linkage specificity) has emerged as the DUB responsible for deactivating the NF-κB signaling downstream of LUBAC through multiple mechanisms discussed in the referred review^108^. Recently, a study revealed a novel regulatory mechanism upstream of OTULIN-mediated NF-κB inactivation that involves non-proteolytic ubiquitylation of OTULIN by the RING-type E3 ligase3 tripartite motif-containing protein 32 (TRIM32)^109^ (Fig. 3b). The authors show that TRIM32 conjugates K63-linked polyubiquitin onto OTULIN which perturbs the interaction between OTULIN and the LUBAC E3 ligase component HOIP (E3 ubiquitin-protein ligase RNF31). As a result, OTULIN can no longer cleave the linear ubiquitin chains generated by LUBAC and NF-κB signaling is maintained.

One of the major regulators of NF-κB pathway activation in response to pro-inflammatory cytokines stimulation is the transforming growth factor-β (TGF-β)-activated kinase 1 (TAK1). Many studies have investigated so far the different PTMs of TAK1, including ubiquitylation and deubiquitylation events that are critical for the regulation of TAK1-mediated NF-kB activation and which are discussed in the suggested review^110^. A more recent study identified the ubiquitin-specific protease 19 (USP19) as a novel DUB that targets TAK1 and acts as a negative regulator of the TNFα and IL1β-triggered NF-kB activation^111^ (Fig. 3b). More specifically, USP19-mediated TAK1 deubiquitylation abrogated TAK1 auto-phosphorylation as well as the formation of the TAK1–TAB2/3 complex, both of which are steps required for efficient NF-kB signaling and thus resulted in attenuated inflammatory response. NF-κB pathway activation is among the major oncogenic signatures in activated B cell-like diffuse large B cell lymphoma (ABC DLBCL) primary tumors. A new molecular mechanism of non-proteolytic ubiquitylation was discovered to play a key role in NF-κB constitutive activation, where the RING E3 ligases cellular inhibitors of apoptosis 1 and 2 polyubiquitylate the B-cell lymphoma/leukemia 10 (BCL10) factor within the CARD11-MALT1-BCL10 (CBM) adapter complex and thus facilitate the recruitment and activation of the established LUBAC/IKK/NEMO signaling axis upstream of NF-κΒ activation^112^ (Fig. 3b). The authors show that second mitochondria-derived activator of caspase (SMAC) mimetics target cIAP1/2 for destruction, thereby suppressing NF-κB and selectively killing ABC DLBCL cells depending on this signaling cascade, providing the perspective of future therapeutic strategies against this lymphoma subtype.

## Non-proteolytic ubiquitylation in cancer signaling

The role of the ubiquitin-proteasome system in cancer is well recognized and is highlighted by the development of proteasome inhibitors as the front line of anticancer treatments currently tested in clinical trials^113^. Nevertheless, the notion that non-K48-linked ubiquitylation can drive oncogenic pathways, has led to a growing demand for the identification of components that could exploit the therapeutic potential of the non-degradative functions of ubiquitylation^114^. Here, we describe some recent examples illustrating new roles of non-proteolytic ubiquitylation in regulating oncogenic and tumor-suppressive signaling pathways in cancer.

**Fig. 4:**
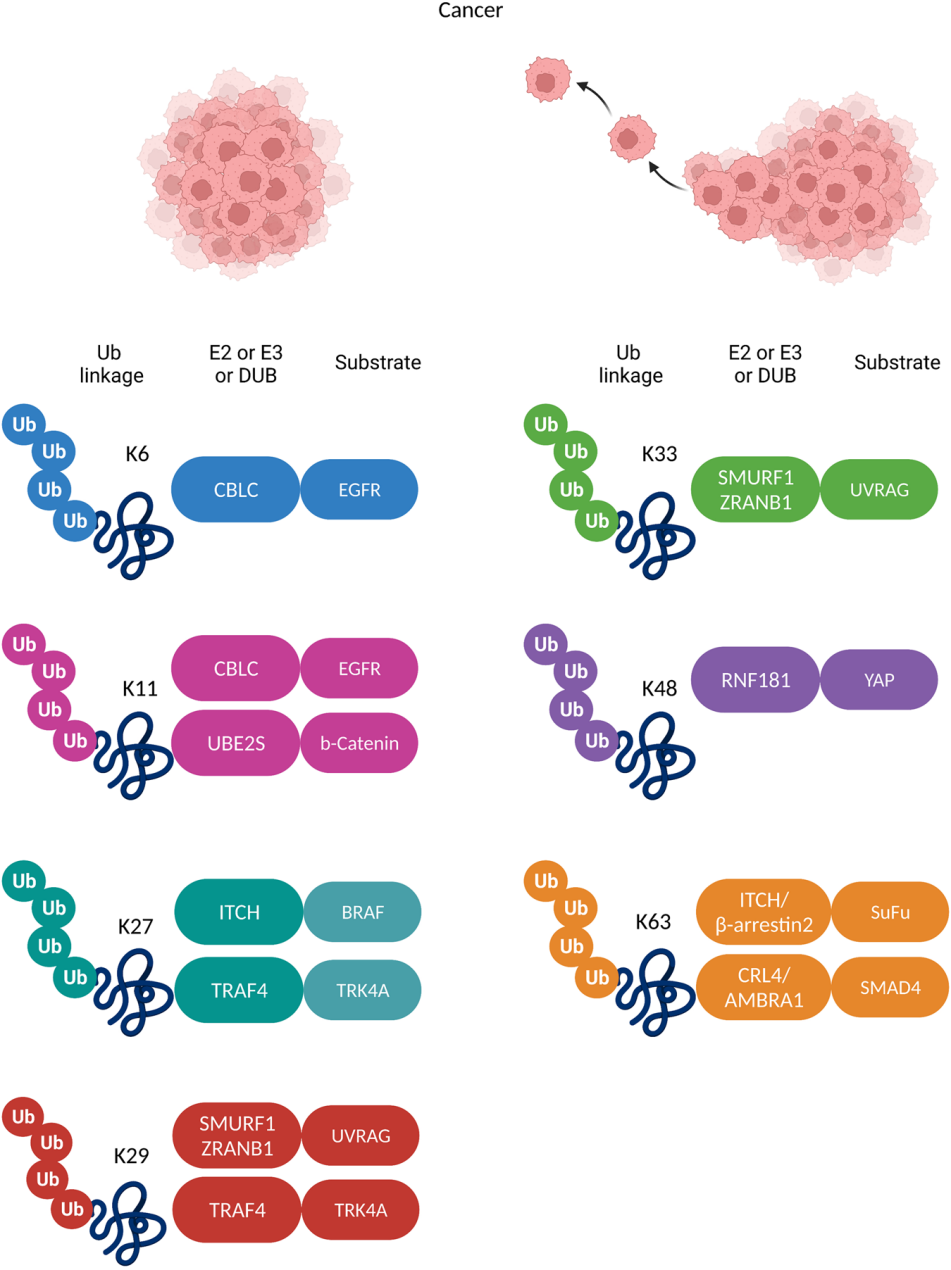
Non-proteolytic ubiquitylation in cancer progression. Examples of reported E2/E3/DUB enzymes (left boxes) and different non-proteolytic Ub signals on the substrate proteins (right boxes) regulating cancer cell proliferation, tumor growth, and metastasis.

Recent findings demonstrate that the autophagy regulator UVRAG (UV radiation resistance-associated) is subjected to non-proteolytic ubiquitylation by the HECT E3 ligase SMURF1 (SMAD specific E3 ubiquitin-protein ligase 1), which promotes autophagosome maturation and inhibits HCC tumor growth in vivo^115^ (Fig. 4). Mechanistically, the SMURF1/UVRAG-dependent autophagic flux is perturbed by counterbalancing modifications on UVRAG including direct deubiquitylation by zinc finger RANBP2-type containing 1 (ZRANB1) and phosphorylation by Casein Kinase 1 alpha 1 (CSNK1A1), thereby providing novel candidates for combined therapeutic intervention against HCC. Suppressor of Fused (SuFu) is a tumor suppressor protein that acts as a critical regulator of the Hedgehog signaling, a conserved pathway with an important role in cerebellar development and tumorigenesis. SuFu is ubiquitylated in a non-proteolytic manner by the HECT E3 ligase ITCH (ubiquitin-protein ligase Itchy homolog) in complex with the adapter protein β-arrestin2, which increases the binding of SuFu to Gli transcription factors and eventually switches off the Hedgehog signaling^116^ (Fig. 4). Considering that medulloblastoma patients often bear SuFu mutations which are insensitive to ITCH ligase activity, it would be worth investigating whether ITCH-mediated ubiquitylation could act as protective mechanism toward the tumor suppressor function of SuFu in cancer types with deregulated Hedgehog signaling. Another example of non-proteolytic ubiquitylation by the ITCH E3 ligase that also acts in concert with phosphorylation events, is proposed to have a pivotal role in melanoma tumorigenesis through the coordination of MER/ERK signaling activation in response to proinflammatory cytokines^117^. The authors demonstrate that cytokine stimulation triggers the activation of ITCH by the C-Jun N-terminal kinase (JNK), which in turn catalyzes the K27-linked polyubiquitylation of the BRAF oncoprotein in order to sustain BRAF activity in transducing mitogenic signals (Fig. 4). Mechanistically, ITCH-mediated BRAF ubiquitylation disrupts its interaction with the 14-3-3 protein, a complex required for maintaining the inhibitory state of BRAF kinase based on structural studies^118^, thus leading to aberrant ERK signaling activation that further promotes melanoma tumor growth.

A recent study provided for the first time evidence that SMAD4 (mothers against decapentaplegic homolog 4), the common signal transducer within the TGF-β and BMP signaling pathways, is subjected to non-proteolytic ubiquitylation by the CRL based on CUL4 (CRL4) and its substrate receptor AMBRA1 (activating molecule in BECN1-regulated autophagy protein 1)^119^ (Fig. 4). The interaction of the CUL4/AMBRA1 complex with SMAD4 enhances its association with the transcriptional co-activator CBP (CREB-binding protein) and subsequently the transcriptional activity of SMAD4, suggesting that this ubiquitylation might create a platform that facilitates the recruitment of essential transcription components to mediate the TGF-β response. AMBRA1 depletion potentiated the metastatic potential of breast cancer cells toward the bones and the lungs in vivo, indicating that the CUL4/AMBRA1-mediated SMAD4 ubiquitylation should be therapeutically investigated in TGF-β-dependent metastatic cancers. In triple-negative breast cancer (TNBC) cellular models, the E3 ligase RING finger protein 181 (RNF181) ubiquitylates and stabilizes YAP, the key effector of the Hippo signaling pathway, potentially protecting it from K48 linked proteolytic ubiquitylation events mediated by the E3 ligases FBW7 and SCF^120^ (Fig. 4). Aberrant activation of the Hippo/YAP signaling downstream of RNF181 promotes migration and invasion phenotypes, suggesting that this modification could serve as a new marker for TNBC patient prognosis.

β-Catenin is the core component of the Wnt/β-Catenin pathway which has established roles in embryonic development and differentiation and its deregulation is strongly linked to colorectal tumorigenesis. Recently, the ubiquitin-conjugating E2S (UBE2S) was found to form a complex with the Cdc27 subunit of the APC/C E3 ligase in order to ubiquitylate β-Catenin and increase it protein stability^121^ (Fig. 4). This modification is non-proteolytic, but seems to antagonize known phosphorylation-induced degradation signals, hence protecting β-Catenin from being recognized and targeted for degradation by the E3 complex Skp1/Cul1/F-box^β-TrCP^. The signaling mediated by UBE2S is proposed to be required for both physiological and pathological processes downstream of β-Catenin, since its ubiquitylation enhances the differentiation of embryonic stem cells into mesoendoderm lineages but also increases the metastatic potential of colorectal cancer cells in vitro and in vivo.

TNF receptor-associated factor 4 (TRAF4) represents another RING E3 ligase which has been suggested to promote cancer cell invasiveness and metastasis through non-proteolytic ubiquitylation in cellular models of prostate cancer^122^. TRAF4 binds to and ubiquitylates the tyrosine receptor kinase A specifically at the cell membrane before its internalization, leading to hyperactivation of its downstream pathways which stimulate the expression of genes associated to invasive phenotypes (Fig. 4). Although the exact mechanism on how TRAF4-mediated ubiquitylation regulates TRKA’s kinase activity remains unclear, the authors propose that ubiquitin attachment might induce conformational changes within the activation loop of TRKA. It would be interesting to investigate if this regulatory mode acts as a more general mechanism in cancers characterized by aberrant tyrosine kinase signaling. Within the context of targeting non-proteolytic ubiquitylation of TRKs in cancer, a recent study on the epidermal growth factor receptor (EGFR) revealed the involvement of the RING E3 ligase CBLC in sustaining an hyperactive EGFR signaling in lung adenocarcinoma both in vitro and in vivo^39^ (Fig. 4). Interestingly, CBLC leads to increased stability and prolonged activation of EGFR and further promotes its trafficking to the nucleus and recycling to the plasma membrane, through competition with another E3 ligase of the same family, known to regulate EGFR turnover^123^. According to the study, CBLC depletion increased the sensitivity of EGFR-mutant lung cancer cells to tyrosine kinase inhibitors (TKIs), which could suggest an alternative strategy to enhance the efficacy of therapeutics in lung cancer patients by the use of combinatory treatments with TKIs and CBLC targeting molecules.

## Non-proteolytic ubiquitylation and metabolism-related diseases

**Fig. 5:**
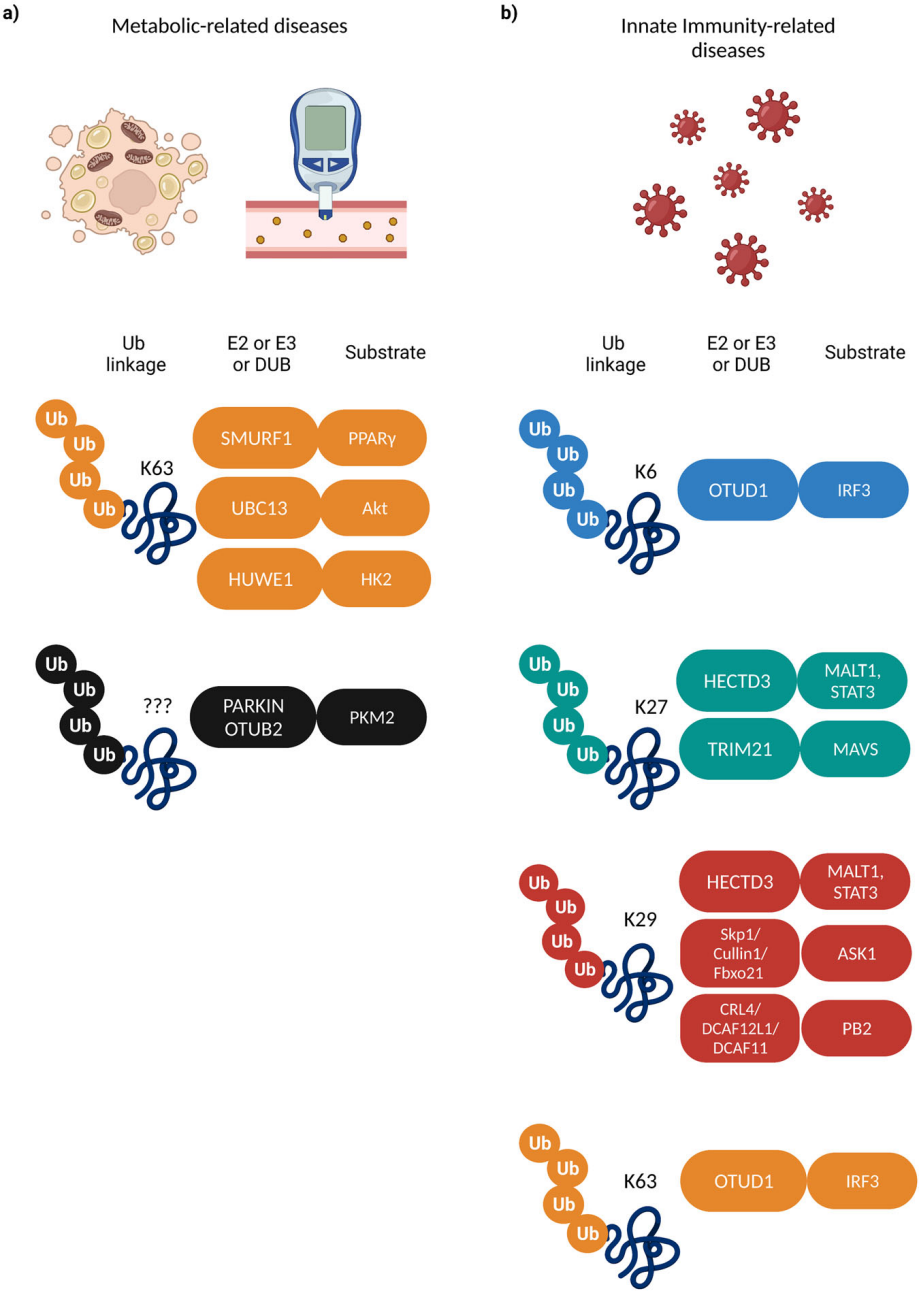
Non-proteolytic ubiquitylation in metabolic and innate immunity related diseases. Examples of reported E2/E3/DUB enzymes (left boxes) and different non-proteolytic Ub signals on the substrate proteins (right boxes) regulating **a** metabolic and **b** innate immunity-related diseases.

Although the role of ubiquitin signaling has not been firmly associated with the development of metabolic disorders, a considerable number of recent findings suggest that non-proteolytic ubiquitylation events are part of the molecular mechanisms underlying metabolic syndromes. Nonalcoholic fatty liver disease (NAFLD) is a chronic liver condition that can severely damage the liver and ultimately lead to cancer^124^. The HECT E3 ligase SMURF1 was found to target the peroxisome proliferator-activated receptor γ (PPARγ), a lipid-sensing nuclear receptor with established role in NAFLD pathogenesis, in order to suppress its transcriptional activity in hepatocytes and balance the PPARγ-mediated fatty acid uptake and lipid synthesis, thereby inhibiting steatosis^125^ (Fig. 5a). Moreover, the fact that the PPARγ antagonist, GW9662, was shown to completely reverse the liver lipid accumulation in mice deficient for SMURF1, suggests that activation of SMURF1-dependent non-proteolytic ubiquitylation could be exploited as a new strategy for NAFLD treatment.

Diabetes is a metabolic disorder characterized by hyperglycemia^126^. There are two main types of diabetes: Type 1 resulting from the autoimmune destruction of insulin-producing pancreatic β cells and type 2 resulting from compromised cellular response to insulin^127^. Several PTMs including ubiquitylation can critically contribute to pancreatic β-cell function and insulin-mediated signal transduction. While the role of proteolytic ubiquitylation in the regulation of diabetes is firmly established, emerging evidence also suggest critical involvement of non-proteolytic ubiquitylation in the development of this disease. Insulin signaling plays a vital role in the regulation of energy homeostasis and its dysregulation is causally linked to diabetes^128^. Protein kinase B (PKB or AKT) is an essential intermediate signaling node, downstream of phosphatidylinositol 3-kinase, in the insulin signaling pathway^129,130^, also implicated in diabetes^131^. It has been reported that the activation of AKT is regulated through K63-linked ubiquitylation upon stimulation with growth factors^132^, suggesting that K63-linked polyubiquitylation plays a critical role in insulin signaling.

Ubiquitin-conjugating enzyme E2 N (UBE2N or Ubc13) is a key E2 enzyme that promotes the formation of non-proteolytic K63-linked polyUb chains^128^. Interestingly, G-protein pathway suppressor 2 (GPS2) is a small multifunctional protein that can directly inhibit UBE2N mediated ubiquitylation^133^ (Fig. 5a). Deletion of UBE2N decreases AKT ubiquitylation and significantly inhibits AKT phosphorylation upon insulin stimulation. In contrast, deletion of GPS2 not only enhances ubiquitylation and activation of AKT but also phosphorylation of the AKT substrate, glycogen synthase kinase 3 beta. Consequently, fat-specific deletion of GPS2 is associated with increased body weight in mice, as a result of lipid accumulation and shift from lipid utilization towards their storage in the adipose tissue^128^. It has been reported that enhanced lipid storing capacity in the adipose tissue can lead to improved systemic insulin sensitivity^134,135^. Hence, it is not surprising that fat-specific deletion of GPS2 results in a positive effect on systemic insulin sensitivity^128^. In conclusion, non-proteolytic ubiquitylation of AKT represents a vital regulatory node in the insulin signaling transduction pathway. In addition, ubiquitin-conjugating E2 enzyme variant 1 (UBE2v1) is a cofactor of Ubc13, able to catalyze Lys63-linked ubiquitylation. Both levels of UBE2v1 and K63-linked ubiquitylated proteins are increased in diabetic patients^136^, suggesting that the UBE2v1-mediated synthesis of K63-linked polyubiquitylation is crucially involved in the development of diabetes.

Metabolic reprogramming is a hallmark of tumorigenesis and it refers to the well-described phenomenon of the Warburg effect in which cancer cells switch to glycolysis even in the presence of oxygen in order to sustain their hyper proliferation rate within a nutrient-deprived tumor microenvironment^137^. An interesting study unraveled a novel signaling axis that regulates cancer metabolism through the non-proteolytic ubiquitylation of hexokinase 2 (HK2), a critical enzyme upstream of many glycolytic pathways, by the HECT-type E3 ligase HECTH9 (or HUWE1)^138^ (Fig. 5a). The authors demonstrated in several models of metabolism-addicted tumors that HECTH9-mediated ubiquitylation orchestrates the trafficking of HK2 to mitochondria and its association with the voltage-dependent anion-selective channel protein at the outer mitochondrial membrane. This ubiquitylation confers to HK2-dependent apoptosis resistance, enhanced glycolysis, and reduced reactive oxygen species generation, ultimately leading to cancer stem cell (CSC) self-renewal and tumor progression. Targeting the HECTH9-HK2 signaling could emerge as an effective strategy to limit CSC expansion and cancer progression in metabolism-addicted tumors. The importance of non-proteolytic ubiquitylation in the metabolic reprogramming of tumor cells has also been confirmed by another recent study which reported a role for the DUB OTUB2 in promoting colorectal cancer progression by elevating the Pyruvate kinase M2 (PKM2) enzymatic activity, subsequently upregulating glycolysis^139^ Upon conditions of nutrient deficiency, OTUB2 co-localizes and interacts with PKM2 in the cytoplasm and blocks its ubiquitylation. Interestingly, this interaction occurs independently of OTUB2 catalytic activity but is instead mediated via competing with the E3 ligase Parkin^140^ for binding to PKM2, highlighting the significance of monitoring the balance between ubiquitylation/deubiquitylation events on key metabolic enzymes as a means to understand how cancer cells respond to metabolic stress (Fig. 5a).

## Non-proteolytic ubiquitylation and innate immunity-related diseases

The role of ubiquitin chains inducing degradation-dependent ubiquitin signaling within the context of the antiviral innate immune response (IIR) has been recently discussed in the suggested review^141^. Here we focus on some recent advances in the regulation of innate immunity during viral infection, governed by non-proteolytic ubiquitylation events. Innate immunity generates a fast non-specific inflammatory response against all types of pathogens by activating the production of interferons (IFNs) and proinflammatory cytokines. Interferon regulatory factor 3 (IRF3) is a transcriptional factor with key functions in the IIR against DNA and RNA viruses and its interplay with the DUB enzyme ovarian tumor domain-containing deubiquitylase (OTUD1) in a non-proteolytic context has gained significant attention. IRF3 is subjected to viral infection-induced K6-linked ubiquitylation which is recognized and cleaved by OTUD1 in a way that impairs the capacity of IRF3 to bind to the promoter region of its target genes.^142^ (Fig. 5b). As a result, the IRF3-dependent production of interferons and pro-inflammatory cytokines is attenuated, leading to increased viral infection sensitivity. Moreover, under inflammatory conditions, IRF3 is deubiquitylated by OTUD1 at K63 ubiquitin chains which inhibits its nuclear translocation and transcriptional activity^143^. The authors also provide evidence that OTUD1 mutations which impair its ability to interact with IRF3, lead to sustained immune response and excessive interferon production that is strongly associated with the development of autoimmune disorders. The above studies highlight the importance of exploiting possible therapeutics targeting the OTUD1-IRF3 signaling axis as a means to ensure immune homeostasis.

One potentially fatal autoinflammatory condition linked to the ubiquitylation pathway is OTULIN-related autoinflammatory syndrome (ORAS), which is caused by a homozygous hypomorphic mutation in the human *OTULIN* gene^108^. Detailed analysis demonstrated that loss-of-function mutations in OTULIN and accumulation of M1-polyUb levels in myeloid cells in a TNF-dependent manner recapitulate the severe phenotypes of inflammation and autoimmunity observed in ORAS patients, which however can be ameliorated by treatment with the anti-TNF neutralizing antibody Infliximab^144^. The important role of OTULIN in restraining inflammation was also suggested by another study in patients suffering from otulipenia due to OTULIN missense mutations that were associated with excessive linear ubiquiylation of target proteins such as NEMO, RIPK1, and TNFR1, resulting in severe inflammation and further confirming OTULIN as a gatekeeper of innate immunity^145^.

IL-17-producing T helper (Th17) cells are a distinct subset of CD4 + T cells whose differentiation and function are controlled by several cytokines and transcription factors and which are considered key determinants in the pathogenesis of autoimmune diseases^146^. Recent evidence in experimental autoimmune encephalomyelitis suggests that HECTD3-mediated non-proteolytic ubiquitylation promotes pathogenic Th17 cell differentiation and function downstream of NF-kB activation^147^. HECTD3 E3 ligase targets two distinct components upstream of T17 cell differentiation, namely MALT1 (mucosa-associated lymphoid tissue 1) and STAT3 (signal transducer and activator of transcription 3) in a non-degradative manner, leading to robust NF-kB signaling activation (Fig. 5b). Moreover, ubiquitylation of both substrates is required for enhancing the expression of RAR-related orphan receptor gamma (RORγt), the master transcription factor controlling Th17 cell identity^148^, further supporting a role for HECTD3 in the development of T17-related autoimmune disorders.

A non-proteolytic role has been suggested for the F-box only protein Fbxo21 in viral-triggered IIR, which until now was a functionally unknown component of the SCF (Skp1–Cul1–F-box protein) complex. Upon viral infection, Fbxo21 ubiquitylates and activates apoptosis signal-regulating kinase 1 (ASK1), a protein with pivotal roles in stress and immune responses, thereby leading to the activation of c-Jun N-terminal kinases (JNK1/2) and p38 MAPKs downstream signaling^149^ (Fig. 5b). Disruption of this modification reduces the production of proinflammatory cytokines and type I interferons and results in attenuated antiviral innate response and enhanced virus replication, suggesting that Fbxo21 might play an indispensable role in regulating innate antiviral response. The first example of K29-linked, non-proteolytic ubiquitylation in the regulation of viral infection was recently described for the PB2 replication protein, an essential component of the viral transcription/replication machinery and a key determinant of host range during influenza A virus (IAV) infection^150^. The authors demonstrate that PB2 undergoes non-degradative ubiquitylation by two distinct RING E3 CRL4 ligase-based multi complexes containing the DDB1 adapter and either DCAF12L1 (D12L1) or DCAF11 (D11) as substrate recognition factors, which however do not complement each other functionally (Fig. 5b). Loss of PB2 ubiquitylation leads to attenuated viral load, indicating that this modification is essential for optimal IAV infection. Further investigation on how CRL4-mediated PB2 ubiquitylation can affect the viral cycle progression would be of great interest and could lead to design of future antiviral therapeutic strategies. The RING-type E3 ligase tripartite motif-containing protein 21 (TRIM21) was shown to display antiviral activity and to facilitate the IIR by exerting non-proteolytic ubiquitylation on a critical component of the innate immune system that is responsible for the downstream transmission of the pathogen recognition signal^151^. Upon conditions of RNA viral infection, TRIM21 is upregulated through the IFN/JAK/STAT pathway, targets the mitochondrial adapter molecule and promotes its interaction with the TBK1, thereby initiating the activation of IRF3 and NF-κB signaling which can then stimulate the production of IFNs and proinflammatory cytokines (Fig. 5b).

## Outlook and conclusions

Deciphering the multifaceted ubiquitin code has proved fundamental for cell biology and for understanding the etiology of several detrimental human diseases. During the last decade, we have witnessed breakthroughs in the development of biochemical tools and cell-imaging technologies that allow us to visualize and monitor the dynamic complexity of the ubiquitin network with unprecedented detail^152^. Based on our existing knowledge, it appears well documented that non-proteolytic ubiquitylation pathways play many important roles in cellular signaling and in the development of various diseases. Unlike ubiquitylation signals that target proteins for their proteasomal degradation, non-proteolytic ubiquitylation mainly regulates proteins’ signaling and trafficking, localization, interplay with other PTMs and interactions. Although the underlying molecular and cellular mechanisms of human pathologies are relatively well understood, the direct links of non-proteolytic ubiquitylation pathways to their etiology are poorly characterized. The use of genetic, and genome-editing approaches in animal disease models are needed to understand the role of non-proteolytic ubiquitylation substrates and their modifying enzymes. Finally, the development of inhibitors targeting the key E3 ligases and DUBs could open interesting experimental possibilities and help in the long-term future to the identification of new therapies against human pathologies.
